# Consumer Perspectives on Antibiotic-Free Animal Products: A Systematic Review Identifying Critical Gaps in Non-Pharmaceutical Intervention Research

**DOI:** 10.3390/ani16010070

**Published:** 2025-12-26

**Authors:** Syed Ayaz Hussain, Syed Raza Abbas, Seung Won Lee

**Affiliations:** 1 Department of Business Management and Commerce, University of Baltistan (UOBS), Skardu 16100, Pakistan; syedayaz72@yahoo.com; 2 Department of Precision Medicine, School of Medicine, Sungkyunkwan University, Suwon 16419, Republic of Korea; shahbaz5.12@skku.edu; 3 Department of Metabiohealth, Institute for Cross-Disciplinary Studies, Sungkyunkwan University, Suwon 16419, Republic of Korea; 4 Department of Artificial Intelligence, Sungkyunkwan University, Suwon 16419, Republic of Korea; 5 Personalized Cancer Immunotherapy Research Center, School of Medicine, Sungkyunkwan University, Suwon 16419, Republic of Korea; 6 Department of Family Medicine, Kangbuk Samsung Hospital, School of Medicine, Sungkyunkwan University, 29 Saemunan-ro, Jongno-gu, Seoul 03181, Republic of Korea

**Keywords:** consumer perception, antibiotic-free, non-pharmaceutical interventions, probiotics, phytogenics, prebiotics, organic acids, enzymes, sustainable animal production

## Abstract

**Background**: The global livestock industry faces pressure to reduce antimicrobial usage while maintaining animal health and productivity. Non-pharmaceutical interventions (NPIs) including probiotics, prebiotics, phytogenics, essential oils, organic acids, and enzymes have emerged as alternatives to antibiotic growth promoters. Commercial success depends on consumer acceptance and willingness to pay (WTP) for products from animals raised using these approaches. **Objective**: This systematic review synthesized peer-reviewed literature examining consumer knowledge, attitudes, perceptions, and WTP toward animal products produced using NPIs or marketed as antibiotic-free (ABF) to identify a critical gap in existing research. **Methods**: Following PRISMA 2020 guidelines, four databases (PubMed, Web of Science, Scopus, and Google Scholar) were searched for peer-reviewed studies published from January 2020 to December 2024. Inclusion criteria encompassed original research examining consumer perspectives toward NPIs or antibiotic-free (ABF) animal products. Narrative synthesis was employed due to study heterogeneity. **Results**: From 847 records, 15 studies met inclusion criteria. A critical finding was that virtually no peer-reviewed research directly examines consumer perceptions of specific NPIs such as probiotics, prebiotics, phytogenics, organic acids, or enzymes as feed additives. The included studies predominantly examined ABF production generally (60%) without specifying alternatives employed. Europe accounted for 80% of studies, while Asia accounted for 20%. Consumer awareness of agricultural antibiotic use was consistently low across contexts. Attitudes toward ABF products were favorable with one study reporting WTP premiums of 18–20%. Health consciousness was the strongest predictor of acceptance. **Conclusions**: The review highlights that while substantial literature exists on ABF products, no studies examine consumer perceptions of specific non-pharmaceutical interventions. Future research should investigate consumer responses to intervention specific labeling and communication strategies.

## Simple Summary

Micro-organisms that resist antibiotics are a growing danger to human health around the world. One major cause is the heavy use of antibiotics in farm animals raised for meat, eggs, and milk. To solve the problem, farmers are now trying natural alternatives like helpful bacteria, plant-based additives, and natural acids added to animal feed. But for such methods to succeed, consumers must be willing to buy and pay more for products made using such approaches. The systematic review looked at scientific studies from 2020 to 2024 to find out what people know and feel about natural alternatives. Fifteen relevant studies were found after searching through 847 research records. The biggest discovery was surprising: almost no research has asked consumers what they think about the specific natural alternatives farmers actually use. Most studies only asked about “antibiotic-free” products in general terms. People knew very little about how antibiotics are used on farms but viewed antibiotic-free products positively and were willing to pay about twenty percent more. Health concerns were the main reason people supported such products. More research is needed to help farmers communicate better with consumers.

## 1. Introduction

Antimicrobial resistance (AMR) represents one of the most pressing public health challenges of the twenty-first century. The World Health Organization (WHO) identifies AMR among the top ten global public health threats with drug-resistant infections causing an estimated 4.95 million deaths worldwide in 2019 [1,2]. Economic projections suggest global GDP losses could reach USD 100 trillion by 2050 if resistance continues uncontrolled [3,4].

**Figure 1:**
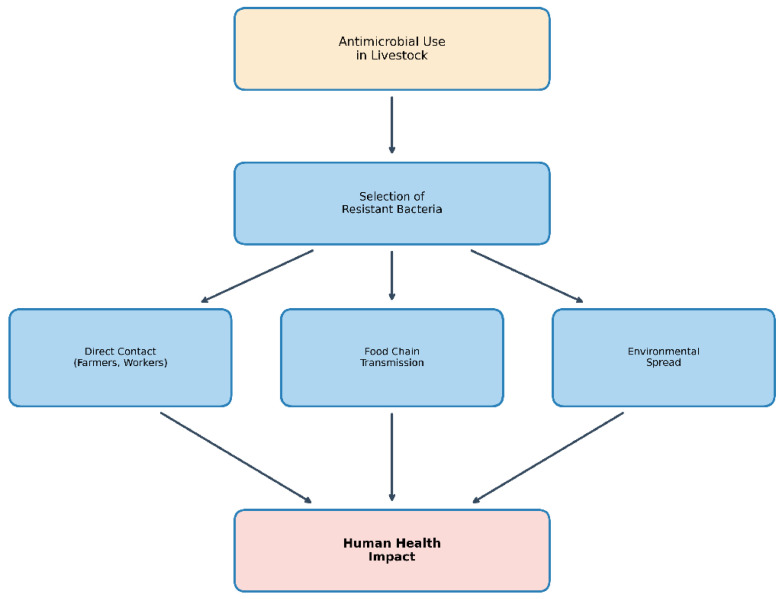
Pathways of antimicrobial resistance transmission from livestock to human populations. Antimicrobial use in livestock selects for resistant bacteria, which can reach human populations through direct contact with agricultural workers, transmission through the food chain via contaminated animal products, and environmental spread through water, soil, and air contamination.

The livestock sector has been identified as a major contributor to AMR with food-producing animals consuming an estimated 73% of all antimicrobials sold globally [5,6]. While therapeutic antimicrobial use remains essential for treating sick animals, prophylactic and growth-promoting applications have been increasingly scrutinized as drivers of resistance [7,8]. Figure 1 illustrates pathways of antimicrobial resistance transmission from livestock to human populations.

In response, regulatory frameworks worldwide have increasingly restricted non-therapeutic antimicrobial use. Sweden banned antimicrobial growth promoters (AGPs) in 1986, followed by the European Union’s complete ban in 2006, the United States’ Veterinary Feed Directive (VFD) in 2017, and China’s comprehensive AGP ban in 2020 [9,10,11]. Key regulatory milestones are summarized in Table 1.

These regulatory changes have driven investment in non-pharmaceutical interventions (NPIs), including probiotics, prebiotics, phytogenics, essential oils, organic acids, and enzymes as alternatives to AGPs [12,13]. These interventions function through mechanisms distinct from antibiotics: probiotics promote a competitive exclusion of pathogens; prebiotics encourage beneficial gut microbes; phytogenics exhibit antimicrobial and antioxidant properties; organic acids lower gastrointestinal pH; and enzymes improve nutrient utilization [14,15].

Consumer acceptance is crucial for the commercial viability of NPI-based production systems. Consumers influence agricultural practices through purchasing decisions affecting producer profitability, market signals determining supply chain requirements, policy advocacy shaping regulations, and social license affecting livestock production acceptability [16,17].

**Table 1:** Global regulatory milestones in agricultural antimicrobial use.

Region/Country	Year	Key Regulatory Action	Region/Country	Year	Key Regulatory Action
Sweden	1986	First country to ban AGPs [18]	European Union	2006	Complete ban on AGPs in animal feed [9]
United States	2017	VFD implemented; AGPs require veterinary oversight [19]	India	2025	Ban on colistin in food-producing animals [20]
South Korea	2011	Complete ban on AGPs [21]	China	2020	Ban on AGPs in commercial animal feed [22]

Abbreviations: AGPs = antimicrobial growth promoters; VFD = veterinary feed directive.

The agriculture sector has experienced both possibilities and challenges primarily as a result of these legislative changes, which have promoted significant investment in non-AGP-based methods of preserving animal productivity and health [23]. Demand for non-pharmaceutical therapies, such as probiotics, prebiotics, phytogenics, organic acids, and enzymes as functional alternatives, has increased due to the shift away from routine antimicrobial use [24]. The animal nutrition sector has undergone a complete transformation as a result of this regulatory environment and rising consumer knowledge of antimicrobial resistance challenges. It has additionally opened up new markets for manufacturers of animal products without the use of antibiotics.

Other market sectors include feed enzymes, which were valued at USD 1.9 billion in 2024 with projections to USD 3.4 billion by 2034 [25], and organic acids, which were valued at roughly USD 2.8 billion in 2024 and projected to reach USD 4.9 billion by 2032 at a compound annual growth rate (CAGR) of 5.8%. Together, these markets are predicted to be worth more than USD 17 billion in 2024, and strong growth will be expected as antimicrobial usage regulations spread throughout the world and consumer preferences become more in favor of goods made from animals that have not been routinely provided antibiotics.

**Figure 2:**
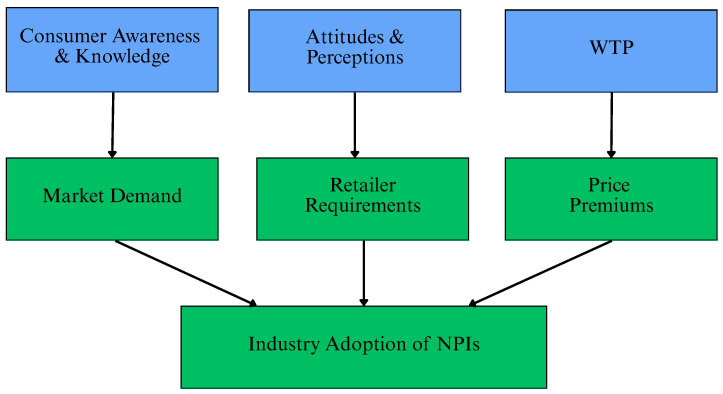
Conceptual pathways linking consumer perspectives to industry adoption of non-pharmaceutical interventions. (WTP: willingness to pay; NPIs: non-pharmaceutical interventions).

Through a variety of channels, consumers are becoming more influential over agricultural practices: purchasing decisions directly impact producer profitability and adoption incentives through WTP premiums; market signals where retailer sourcing decisions and supply chain requirements are determined by aggregated consumer preferences, social license where consumer attitudes impact the wider social acceptability of livestock production practices [17]. The conceptual processes through which consumer attitudes impact the industry adoption of NPIs can be seen in Figure 2.

### Rationale and Objectives

Despite the growing importance of NPIs in animal production, no systematic review has comprehensively examined consumer attitudes toward these specific interventions. Previous reviews have addressed consumer attitudes toward organic products [26], antibiotic use in animal production [27], or novel proteins [28], but none has focused specifically on NPIs as antibiotic alternatives.

This systematic review addresses this gap through five objectives:

1. Synthesize peer-reviewed literature examining consumer perspectives toward NPIs and antibiotic-free (ABF) animal products;
2. Characterize the geographic, methodological, and thematic scope of existing research;
3. Assess consumer knowledge, attitudes, and willingness to pay (WTP);
4. Identify factors influencing acceptance; and
5. Establish priorities for future research.

## 2. Materials and Methods

### 2.1. Protocol and Guidelines

This systematic review was conducted and reported following the Preferred Reporting Items for Systematic Reviews and Meta-Analyses (PRISMA) 2020 guidelines [29] (Supplementary Materials). The review protocol was developed a priori and specified the research questions, search strategy, eligibility criteria, data extraction procedures, quality assessment methods, and synthesis approach.

### 2.2. Eligibility Criteria

We included peer-reviewed publications that examined consumer-related outcomes associated with non-pharmaceutical feed additives (e.g., probiotics, prebiotics, synbiotics, phytogenics/plant extracts/essential oils, organic acids, enzymes) and/or antibiotic free or reduced-antibiotic animal production. Eligible outcomes included consumer knowledge/awareness, attitudes/perceptions, acceptance, trust, purchase intention, WTP, preference/choice behavior, and related market or policy perceptions.

#### 2.2.1. Inclusion Criteria

We included (i) primary empirical studies reporting original consumer data (e.g., surveys, experiments, choice/conjoint studies, interviews/focus groups, mixed-methods) alongside (ii) evidence-synthesizing papers (narrative reviews, systematic reviews, meta-analyses) and policy reviews/analyses that explicitly synthesized or evaluated consumer perspectives relevant to the topic.

The inclusion of contextual reviews and modeling studies alongside empirical consumer research was a deliberate methodological choice. This mixed-evidence approach was adopted because (1) the field of consumer perceptions toward NPIs is nascent, requiring broader contextualization; (2) contextual papers provide essential background on production systems that shape the market environment consumers respond to; and (3) this approach enables the identification of disconnects between production practices and consumer awareness. However, our synthesis prioritizes findings from empirical consumer studies explained later in Section 3.3 providing supplementary evidence on the broader sustainable production landscape. This review therefore functions as a mixed-evidence systematic review following PRISMA 2020 guidelines.

#### 2.2.2. Exclusion Criteria

We excluded commentaries, editorials, protocols, conference abstracts, theses/dissertations, non-peer-reviewed gray literature, and papers not focused on consumer outcomes or not related to the target interventions/production claims. Studies addressing only technical/biological performance (e.g., animal growth or microbiology) without any consumer component were excluded.

### 2.3. Information Sources

Four electronic databases were systematically searched to ensure comprehensive coverage of the literature:

1. PubMed: primary database for biomedical and life sciences literature;
2. Web of Science Core Collection: multidisciplinary database with strong coverage of agricultural and food sciences;
3. Scopus: comprehensive multidisciplinary database with strong social science coverage;
4. Google Scholar: supplementary search for grey literature identification and citation tracking.

### 2.4. Search Strategy

The search strategy used a single combined Boolean string that integrated three conceptual domains (consumers, non-pharmaceutical feed interventions, and animal products). Truncation (*) and phrase searching (“ ”) were applied where appropriate.

**Combined search string (common format):**

(consumer* OR public OR perception* OR attitude* OR knowledge OR awareness OR acceptance OR “willingness to pay” OR WTP OR “purchase intention*” OR preference* OR behavior* OR behaviour* OR choice* OR demand) AND (probiotic* OR prebiotic* OR synbiotic* OR phytogenic* OR “essential oil*” OR “plant extract*” OR botanical* OR “organic acid*” OR enzyme* OR “antibiotic-free” OR “antibiotic free” OR “raised without antibiotics” OR “no antibiotics” OR “reduced antibiotic*” OR “antibiotic alternative*” OR “natural feed additive*” OR “feed additive*” OR “growth promoter alternative*”) AND (poultry OR chicken OR broiler OR turkey OR duck OR pork OR pig OR swine OR beef OR cattle OR livestock OR meat OR “animal product*” OR “food animal*” OR egg* OR dairy OR milk)

### 2.5. Study Selection Process

Study selection followed a systematic two-stage process.

#### 2.5.1. Stage 1: Title and Abstract Screening

Two reviewers (initials) independently screened all titles and abstracts against inclusion criteria. Each record was classified as one of the following:

- Include: clearly meets inclusion criteria;
- Exclude: clearly fails to meet one or more inclusion criteria;
- Uncertain: requires full-text review to determine eligibility.

Disagreements between reviewers were resolved through discussion. Records classified as “Include” or “Uncertain” by either reviewer proceeded to full-text screening.

#### 2.5.2. Stage 2: Full-Text Assessment

Full texts of potentially eligible studies were retrieved and independently assessed by two reviewers. Reasons for exclusion were documented for all excluded full texts. Disagreements were resolved through discussion or consultation with a third reviewer.

**Figure 3:**
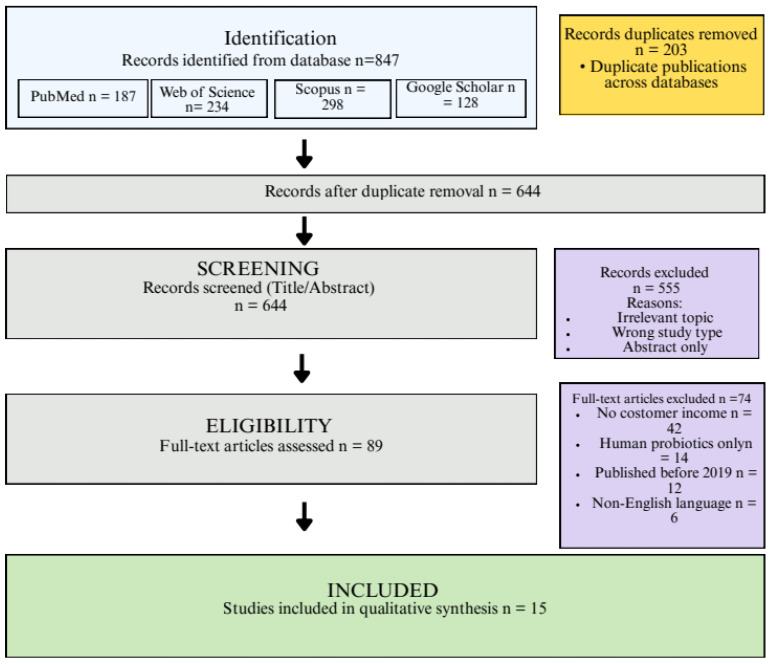
PRISMA flow diagram of the study identification and selection process.

Figure 3 presents the PRISMA flow diagram documenting the study selection process.

### 2.6. Data Extraction

Data were extracted from included studies using a standardized extraction form developed for this review. Table 2 details the data items extracted.

### 2.7. Quality Assessment

In addition to factors unique to consumer research methods, criteria modified from the Mixed Methods Appraisal Tool (MMAT) version 2018 [30] were used to evaluate the quality of the study. The quality assessment criteria can be seen in Table 3.

### 2.8. Data Synthesis

Quantitative meta-analysis was found to be inappropriate due to significant heterogeneity across study designs, demographics, interventions, and outcome measures.

The synthesis procedure included the following elements:

1. Developing a preliminary synthesis: tabulating study characteristics and findings;
2. Exploring relationships: identifying patterns, similarities, and differences across studies;
3. Assessing robustness: evaluating consistency of findings and methodological quality;
4. Integrating evidence: synthesizing findings into coherent thematic summaries.

Five concept domains had been employed to compile the findings:

1. Consumer knowledge and awareness;
2. Attitudes and perceptions;
3. WTB;
4. Factors influencing acceptance;
5. Geographic and demographic variations.

## 3. Results

### 3.1. Study Selection Overview

The 847 records have been identified in four databases by the scientific search. Of these, 644 distinct items went through screening by title and abstract after duplicates (*n* = 203) were eliminated. Afterwards, 89 articles were left for full-text evaluation after 555 were eliminated for failing to meet inclusion criteria. A total of 15 research studies were included in the final synthesis after 74 publications were dismissed following a comprehensive full-text examination.

### 3.2. Critical Finding: Identification of a Major Research Gap

During the systematic search process, a crucial and surprising discovery was made: there has been essentially no peer-reviewed consumer research in the scientific literature that specifically examines attitudes toward named NPIs (probiotics, phytogenics, prebiotics, organic acids, and enzymes) as feed additives in animal production.

We anticipated finding studies having evaluated consumer attitudes about specific categories of interventions, similar to how consumers might assess “probiotic-fed chicken” or “phytogenic-enhanced pork;” however, none of these have been identified. The broad application of these interventions in industry practice and the almost nonexistent analysis of consumer awareness or approval of particular treatments in consumer research fundamentally contradict one another.

None of the 15 included studies specifically addressed any of the previously mentioned interventions; instead, they fell into three different categories:

1. ABF/reduced-antibiotic studies (*n* = 9; 60%): These examined consumer perspectives toward products marketed broadly as ABF or raised without antibiotics without specifying which alternative interventions enabled such claims.
2. Novel alternative feed ingredient studies (*n* = 4; 26.7%): These examined the consumer acceptance of products from animals fed innovative alternative ingredients distinct from the established NPIs that were the focus of this review.
3. Natural/sustainable production studies (*n* = 2; 13.3%): These addressed broader consumer preferences for natural or sustainable production methods without reference to specific interventions.

**Figure 4:**
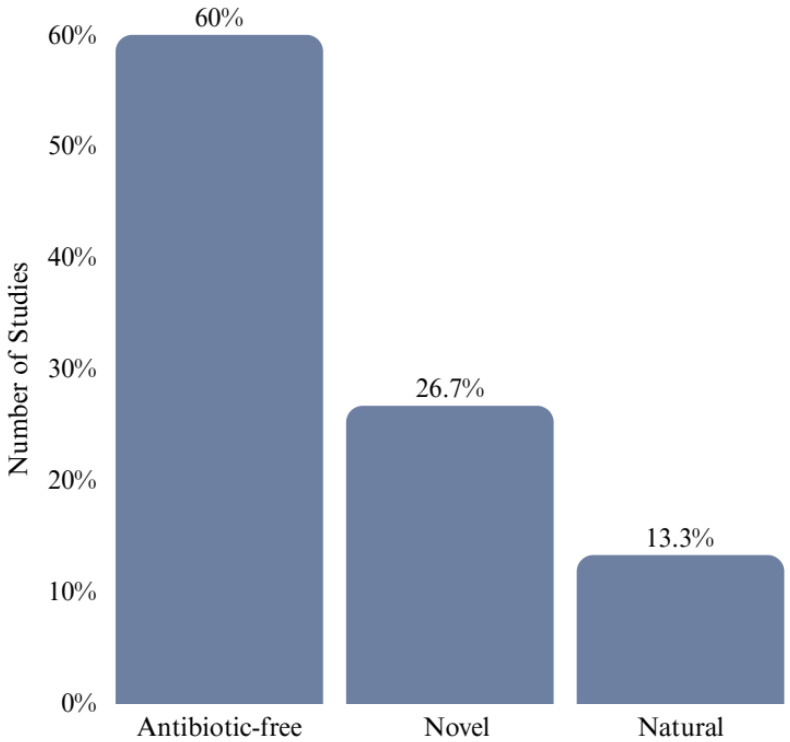
Distribution of included studies by intervention category.

Figure 4 illustrates the distribution of included studies by category.

### 3.3. Characteristics of Included Studies

Included studies were geographically diverse but unevenly distributed. Most evidence originated from Europe (*n* = 12), followed by Asia (*n* = 3), with single studies representing North America (USA) and Oceania (Australia), and one multi-country study spanning the UK and Ireland. Because some studies included multiple contexts or were classified across more than one geographic label, regional categories may overlap; therefore, Table 4 provides the definitive breakdown used in this review.

Across the included studies, cross-sectional surveys were the dominant design (*n* = 10; 66.7%), while discrete choice experiments (DCEs) were also common (*n* = 3; 20.0%); only one study used an experimental auction and one used a quasi-experimental information intervention (each *n* = 1; 6.7%). Data collection was primarily online (*n* = 12; 80.0%) with fewer in-person studies (*n* = 2; 13.3%) and rare mixed-mode approaches (*n* = 1; 6.7%). Sample sizes ranged widely: four studies included fewer than 500 participants, four enrolled 500–999, five enrolled 1000–1999, and two exceeded 2000 participants. Analytically, all studies reported descriptive statistics (*n* = 15; 100.0%), most applied regression-based modeling (*n* = 12; 80.0%), and smaller subsets used structural equation modeling (CVM) (*n* = 3; 20.0%), latent class analysis for segmentation (*n* = 2; 13.3%), and choice modeling approaches (*n* = 3; 20.0%).

In terms of product focus, poultry products were most frequently examined (*n* = 8; 53.3%), typically including chicken breast, whole chicken, or poultry meat in general. Pork products were the second most common category (*n* = 4; 26.7%), which were followed by studies that addressed multiple or general meat products without restricting to a single species (*n* = 2; 13.3%). Beef was least represented, appearing in only one study (*n* = 1; 6.7%).

Table 5 presents comprehensive characteristics of all 15 included studies.

#### Quality Assessment Results

Quality assessment was conducted for all included studies using criteria adapted from the Mixed Methods Appraisal Tool (MMAT) version 2018 [43]. Among the seven empirical consumer studies in Table 6, Panel A), quality ratings were generally moderate to high. All studies clearly stated research objectives (Q1: 7/7) and used appropriate study designs (Q2: 7/7). Sampling strategies were adequately described in most studies (Q3: 6/7), though sample representativeness varied (Q4: 5/7 acknowledged limitations). Measurement instrument validity was demonstrated in studies using established scales (Q5: 5/7), while statistical analyses were appropriate across studies (Q6: 7/7). Consideration of confounding factors (Q7: 4/7) and acknowledgment of limitations (Q8: 6/7) were inconsistent. The contextual reviews and modeling studies (Panel B) were assessed for methodological rigor appropriate to their study type; all met basic quality thresholds for inclusion. Overall, the empirical evidence base was of moderate quality with limitations primarily related to convenience sampling, geographic concentration, and hypothetical bias in WTP elicitation methods.

### 3.4. Consumer Knowledge and Awareness

Across the included literature, consumer knowledge about antibiotic use in animal production was consistently limited regardless of geographic setting. Studies reported low objective knowledge in China with many participants unable to correctly identify antibiotic-use practices or relevant rules [43]. In Iran, baseline awareness was additionally low, but in that setting, higher awareness had been linked to outcomes related to consumption [24]. Limited comprehension was additionally noticed in the UK and Ireland, indicating that knowledge gaps are not solely specific to any one area [46].

The difference between what customers think that they comprehend and what they can demonstrate when evaluated was a recurrent theme. Subjective confidence frequently surpassed objective knowledge, according to several research studies, resulting in a knowledge confidence gap that may lead to a greater reliance on straightforward cues rather than precise information [43]. Terminology confusion had been prevalent with important concepts like “antimicrobial resistance,” “ABF” and “raised without antibiotics” often misrepresented or confused [46]. In addition, regulatory awareness was limited; for example, UK evidence showed that few consumers recognized existing restrictions governing antibiotic use in production systems [34].

Evidence also suggests that awareness is often higher for *labels* than for the underlying meaning of those claims. In the United States, consumers showed moderate recognition of ABF labeling, but understanding of the implications and what such labels do (and do not) guarantee remained limited [35]. This pattern reinforces the idea that consumers may respond to the presence of a claim while lacking the background knowledge needed to interpret it accurately or compare it to alternative claims across products [34].

This review identified a major gap in the evidence base: no included study directly examined consumer awareness of specific non-pharmaceutical interventions used in animal feed, such as probiotics, prebiotics, phytogenics, organic acids, or enzymes. This is notable because consumers may be familiar with some of these concepts in human nutrition, yet the available consumer research does not test whether people know these interventions are used in animal production or how they interpret intervention-specific claims. The synthesis highlights that prior awareness had been typically poor and that attitudes frequently depended on information provision when research addressed innovative alternative elements like insects or algae, highlighting the crucial function of communication when technical literacy is inadequate [46].

### 3.5. Consumer Attitudes and Perceptions

Consumer perceptions of ABF and reduced-antibiotic animal products were generally favorable throughout the included trials with these items considered as a better option than meat that is produced conventionally. ABF claims were often perceived as indicating a “better” product across settings, which resulted in generally positive assessments and increased reported desire to select such options when available [35].

Perceived personal benefit was a dominant attitudinal motivation. ABF products have consistently been linked to increased safety and healthfulness, particularly through decreased worries about antibiotic residues and dangers associated with antimicrobial resistance [43]. At the same time, ABF was frequently linked with broader quality cues, such as expectations of superior flavor or overall quality and judgments of greater “naturalness” or lower processing, that further reinforced favorable attitudes [34,46].

Additionally, a number of research studies indicated that ABF labeling implies social and ethical implications that extend beyond immediate health. Although there was relatively little and occasionally indirect evidence on environmental views, many consumers associated ABF offers with presumptions about improved animal welfare and, to a lesser extent, more sustainable production [35]. Crucially, consumers’ basic knowledge gaps and terminology confusion shaped these positive associations, indicating that ABF can serve as a general heuristic even in situations where individuals have been unable to precisely define antibiotic use practices or regulations [34].

Attitudes toward new feed-related developments have been more conflicted and frequently started with opposition in comparison with ABF products. Unfamiliarity, perceived “disgust” reactions, and safety concerns were frequently cited as reasons for initial reluctance; however, studies showed that acceptance could improve when consumers received clear information and reassurance regarding safety, benefits, and production practices [32]. This trend shows that while antibiotic reduction measures are frequently approved right away, new intervention paths might need to be explicitly communicated and framed for the purpose of enhancing consumer acceptance [31].

**Figure 5:**
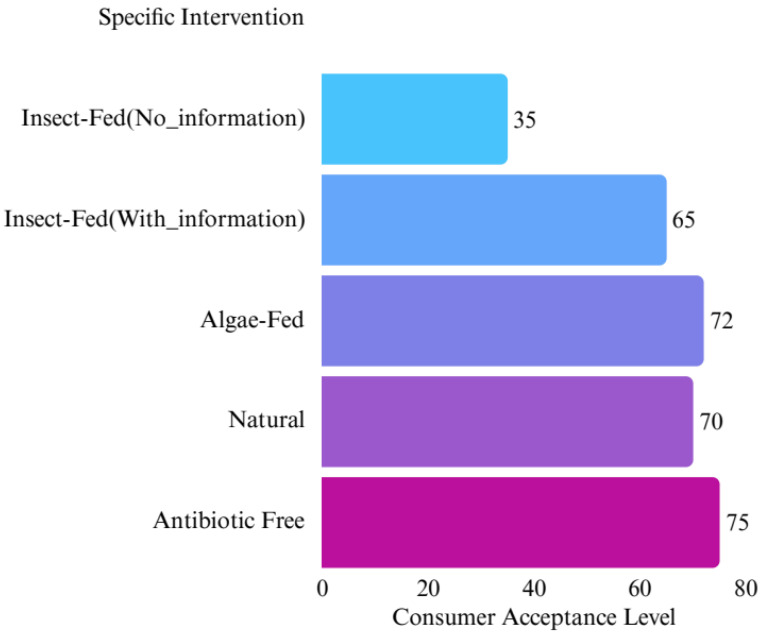
Comparative consumer acceptance levels across intervention types (synthesized estimates from included studies).

The attitudes of various intervention types are compared in Figure 5.

### 3.6. Willingness to Pay

#### 3.6.1. WTP for Antibiotic-Free and Sustainable Products

Although estimates varied significantly based on methodology, product type, and regional context, WTP premiums for ABF and sustainably produced animal products were found to be consistently favorable across the included research. The WTP results from the empirical consumer research in this review are compiled in Table 7.

Jahanabadi et al. [44], one of the research studies looking especially at ABF products, discovered that Iranian consumers had been prepared to pay an 18–20% premium for ABF chicken meat utilizing contingent valuation methods (CVMs). In their sample of 525 respondents, education level, household income, and knowledge of antibiotic resistance issues were found to be significant predictors of WTP. Similarly, although they could not provide precise premium estimates, Mohammadi et al. [24] determined that among 360 Iranian consumers, health consciousness and food safety concerns had been important factors influencing the consumption of ABF poultry.

Gross et al. [31] carried out a mixed sensory and discrete choice experiment with 155 German consumers to assess WTP for animal welfare pork in the context of sustainable production and animal welfare. According to their findings, hedonic liking and WTP were strongly impacted by knowledge about animal welfare procedures with organic certification fetching higher premiums than particular welfare labels. A cross-national survey in Germany, Italy, and the United States (N = 2670) has been carried out by Busch et al. [32] to investigate perceptions of antibiotic use in cattle production. Although they did not provide precise WTP estimates, their results showed that consumers believed antibiotic use posed health hazards and were doubtful of the industry’s ethical methods, indicating a latent demand for ABF substitutes.

By using focus groups with 36 customers in the UK and Ireland, Regan et al.’s qualitative study [46] revealed supplementary insights. Participants showed a high level of awareness and worry about antimicrobial resistance but had relatively little knowledge of the agri-food sector’s transmission mechanisms. This suggests that providing information could improve acceptance and maybe even WTP for goods that do not contain antibiotics.

#### 3.6.2. Methodological Influences on WTP Estimates

The WTP evidence in this review is limited in scope and geographic coverage. The most precise WTP estimate comes from a single study: Jahanabadi et al. [44] found that Iranian consumers were willing to pay an 18–20% premium for ABF chicken using CVMs. Other included studies provided qualitative or indirect evidence regarding consumer WTB premiums but did not quantify specific percentages. Notably, the European studies comprising 80% of the included research focused primarily on attitudes, awareness, and acceptance rather than precise WTP quantification. This geographic and methodological limitation means that WTP estimates cannot be generalized broadly, and future research should employ incentive-compatible methods across diverse geographic contexts.

Survey-based research, like that conducted by Adam and Bruce [33], Mohammadi et al. [24], and Zhou et al. [43], focuses more on attitudes, intentions, and acceptance than on precise monetary valuations. Although this method restricts the direct comparability of WTP estimates, it additionally provides insightful information about the demographic and psychological aspects of consumer preferences. German consumers demonstrated the most critical attitudes about antibiotic usage in cattle, followed by Italian and American respondents, as determined by Busch et al.’s cross-national design [32].

### 3.7. Factors Influencing Consumer Acceptance

Several factors impacting consumer acceptance of ABF and sustainably produced animal products have been established by synthesizing the collected studies. These elements fall under four categories: demographics, trust dimensions, knowledge and information, and health-related issues. A comprehensive review of these factors can be found in Table 8.

#### 3.7.1. Health-Related Factors

In all of the included investigations, health consciousness has been shown to be one of the best and most reliable indicators of acceptance. Using the extended TPB framework, Zhou et al. [43] discovered that among 1123 Chinese customers, health consciousness demonstrated a substantial impact on purchase intention toward meat produced without preventive antibiotic use. Health consciousness has been shown to be a significant factor in determining the consumption of ABF poultry in Iran by Mohammadi et al. [24]. Concerns about food safety were also consistently linked to a preference for ABF goods because consumers who felt that conventional production methods posed larger dangers exhibited a stronger desire for alternatives [44,46].

#### 3.7.2. Knowledge and Information

Knowledge performed a complicated and varied function in influencing customer acceptability. According to Adam and Bruce [33], consumers in the UK showed little understanding of the use of antibiotics in agriculture with almost 50% of respondents saying they “don’t know” when asked about antimicrobial resistance and how it relates to animal production. This result indicates that there is a lot of room for information-based treatments to increase acceptability. In a comparable direction, Regan et al. [46] found that Irish and UK consumers demonstrated high awareness but low comprehension of AMR transmission from the agri-food sector, suggesting that awareness alone might prove insufficient without a better grasp of the concerns involved.

Providing information has been shown to have proved a potentially effective strategy for improving acceptance. Gross et al. [31] showed that sensory evaluation and WTP for pork products were considerably impacted by knowledge of animal welfare procedures. The review papers which make up part of this synthesis [34] highlighted how crucial good science communication is to closing the knowledge gap between production methods and consumers.

#### 3.7.3. Trust Dimensions

Consumer reactions were significantly influenced by trust in different individuals and institutions. According to Busch et al. [32], consumers in Germany, Italy, and the US believed that the cattle industry was not employing antibiotics responsibly, which is a sign of low industry trust. The qualitative results of Regan et al. [46], in which participants expressed doubt regarding the reasons for antibiotic use in farming, mirrored this skepticism.

Additionally, there seemed to be more constant positive trust in certification and labeling systems. Consumers preferred accessibility and clear information regarding antibiotic use procedures, according to Adam and Bruce [33], who suggested that reliable third-party certification could improve acceptability. Numerous studies have emphasized the significance of regulatory monitoring with government-backed norms typically being seen more favorably than industry self-regulation.

#### 3.7.4. Demographic Characteristics

Different research studies revealed different connections between demographic characteristics and acceptance. Consumer awareness and the acceptability of ABF and sustainable production practices were consistently found to be positively correlated with education [24]. Income was also linked to acceptance, which probably reflects rich customers’ increased health consciousness and capacity to pay premiums [44].

The impact of age varied considerably between research and situations. Younger generations in Hungary have distinct worries about livestock production than older farmers and customers, according to Farkas et al. [40]. When gender disparities were analyzed, it was found that although this pattern was not consistent across all research, female customers frequently reported greater worry about food safety and animal welfare issues.

## 4. Discussion

This systematic review aimed to synthesize peer-reviewed literature examining consumer perspectives toward NPIs in animal production and ABF products while identifying critical gaps in existing research. The most significant finding is the virtual absence of consumer research specifically examining attitudes toward named NPIs, which is a gap that has important implications for industry communication strategies and future research priorities. The included studies instead addressed consumer perspectives on ABF production generally or broader sustainability considerations, providing valuable but indirect evidence regarding the commercial viability of NPI-based production systems.

Two related theme areas were covered by the included studies. Studies from Zhou et al. [43], Jahanabadi et al. [44], Mohammadi et al. [24], Adam and Bruce [33], Regan et al. [46], and Busch et al. [32] represented the first theme. Although consumers’ awareness of real practices and antimicrobial resistance mechanisms continues to be limited, these studies repeatedly showed that consumers express worry about antibiotic usage within cattle farming and reveal WTP premiums for ABF products. The second theme addressed broader sustainability and animal welfare considerations in livestock production, which were examined through both empirical consumer research [40] and review articles synthesizing evidence on environmental impacts and production system sustainability.

A significant finding across the empirical consumer studies is the consistent gap between consumer awareness and understanding. Adam and Bruce [33] found that approximately 50% of UK consumers responded “don’t know” to questions about antibiotic use in agriculture, while Regan et al. [46] observed high awareness but low comprehension of antimicrobial resistance transmission pathways among Irish and UK consumers. This pattern suggests that while consumers are broadly aware of antibiotic-related concerns in food production, they lack the detailed knowledge necessary for fully informed decision making. The cross-national study by Busch et al. [32] further revealed that consumers across Germany, Italy, and the United States perceive health risks from antibiotic use in livestock and are skeptical about whether the industry acts responsibly, indicating a trust deficit that may influence purchasing behavior and policy preferences.

### 4.1. Interpretation and Theoretical Implications

The identified gap between the industry adoption of NPIs and consumer research on these interventions warrants particular attention. While probiotics, prebiotics, phytogenics, organic acids, and enzymes are now widely used in commercial animal production globally, consumers appear to encounter these interventions only through outcome-based claims (e.g., “ABF,” “raised without antibiotics”) rather than intervention-specific information. This pattern may reflect industry assumptions that technical production inputs are not meaningful to consumers, as noted by reviewers of this field. However, this assumption remains empirically untested. Given that some NPIs (particularly probiotics) have established consumer recognition in human nutrition contexts, there may be opportunities for intervention-specific marketing that leverages existing consumer familiarity. Alternatively, outcome-based messaging may indeed be more effective, as it communicates benefits rather than production methods. Resolving this question requires direct empirical investigation comparing consumer responses to intervention-specific versus outcome-based claims.

The findings from this review can be interpreted through the lens of credence quality theory, which provides a useful framework for understanding consumer behavior toward production attributes that cannot be verified through direct inspection or consumption experience. ABF production and sustainable farming practices represent classic credence qualities because consumers must rely entirely on external information sources, labels, and certifications to evaluate these attributes. This theoretical perspective explains several consistent findings across the included studies, including the importance of trusted labels and third-party certification [31,33], consumer reliance on outcome-based claims rather than technical production specifications [43], and the significant impact of information provision on attitudes and WTP [31,46].

The consistent finding that information provision can substantially improve consumer attitudes aligns with dual-process models of cognition from social psychology. When consumers lack relevant information, they tend to rely on heuristic processing using simple cues such as price, brand familiarity, or general category labels. However, when provided with meaningful information about production practices and their implications, consumers can engage in a more systematic evaluation of product attributes. Gross et al. [31] demonstrated this pattern clearly, showing that information about animal welfare practices significantly affected both sensory evaluations and WTP for pork products among German consumers. Similarly, Regan et al. [46] found that exposure to risk information about antimicrobial resistance influenced how consumers made sense of agricultural antibiotic use, though the effects were complex and mediated by pre-existing beliefs and social representations.

Health consciousness emerged as the most consistent predictor of acceptance across the included studies, supporting health belief models that emphasize perceived susceptibility and severity as drivers of health-protective behaviors. Zhou et al. [43] found that health consciousness significantly influenced purchase intention toward meat produced without preventive antibiotic use, operating through attitudes within the TPB framework. Mohammadi et al. [24] and Jahanabadi et al. [44] similarly identified health consciousness and food safety concerns as key determinants of antibiotic-free product acceptance in Iranian consumer samples. This consistent pattern suggests that marketing and communication strategies emphasizing health benefits may be particularly effective for promoting sustainable production practices.

The review articles examining livestock sustainability [34,42,45] highlight an important disconnect between scientific evidence on production system sustainability and consumer perceptions. While these reviews demonstrate that improved animal health through vaccination, disease prevention, and management practices can enhance both economic and environmental sustainability, consumer understanding of these relationships appears limited. Farkas et al. [40] documented how structural changes in Hungarian livestock farming, including farm concentration and the decline of small-scale production, have affected rural communities in ways that may not align with consumer preferences for “traditional” or “local” production. This disconnect between technical sustainability metrics and consumer values represents a communication challenge for the livestock sector.

### 4.2. Practical Implications

The findings from this systematic review carry important implications for industry stakeholders, policymakers, and researchers working to promote sustainable livestock production while meeting consumer expectations. For industry stakeholders, current evidence suggests that outcome-based messaging emphasizing ABF or reduced-antibiotic production is understood and valued by consumers, while more technical claims about specific production interventions may not resonate without substantial consumer education. The consistent finding that health consciousness drives acceptance suggests that marketing strategies should emphasize health benefits and food safety rather than technical production details. Building consumer trust emerges as a critical priority given the low confidence in industry responsibility documented by Busch et al. [32], and third-party certification from credible organizations may help address this trust deficit [31,33].

For policymakers, the review findings support continued regulatory efforts to reduce antimicrobial use in agriculture, as consumer acceptance of ABF production appears robust across diverse geographic and cultural contexts. However, the substantial knowledge gaps documented across studies suggest that public education initiatives could enhance informed consumer choice and support for sustainable production policies. Regan et al. [46] specifically noted that consumers displayed misconceptions about antimicrobial resistance transmission from the agri-food sector, indicating opportunities for targeted educational interventions. Given consumer confusion about terminology and label claims documented by Adam and Bruce [33], clear and consistent labeling standards would benefit both consumers seeking to make informed choices and producers seeking to communicate their practices effectively.

The economic analyses and modeling studies included in this review [42,47] provide evidence that sustainable production practices can be economically viable, though consumer WTP premiums remains an important factor in market viability. Jahanabadi et al. [44] found that Iranian consumers were willing to pay 18–20% premiums for ABF chicken with education and income as significant predictors of WTP. These findings suggest that premium market segments exist for sustainably produced animal products, though price sensitivity may constrain broader market adoption. The regional analysis by Di Vita et al. [41] identified substantial variation in sustainability practices across European beef farming regions, suggesting that locally adapted approaches may be necessary rather than uniform policy prescriptions.

### 4.3. Limitations and Future Research Directions

This systematic review has several limitations that should be considered when interpreting the findings. The included studies varied substantially in methodology, geographic context, and specific focus, limiting the potential for quantitative meta-analysis and requiring narrative synthesis approaches. The concentration of empirical consumer research in a limited number of countries, particularly Iran, the UK, Germany, and the United States, raises questions about generalizability to other cultural and economic contexts. Several included studies relied on convenience sampling or online panels, which may not fully represent broader consumer populations. Additionally, the predominance of hypothetical valuation methods in WTP studies suggests that actual market premiums may be lower than reported estimates due to the well-documented hypothetical bias in stated preference research.

The review also revealed important gaps in the existing literature that represent opportunities for future research. Most notably, there is limited research examining consumer responses to specific NPIs such as probiotics, prebiotics, phytogenics, and organic acids that are increasingly used in commercial animal production to support animal health and reduce antibiotic dependence. While consumers appear to respond positively to outcome-based claims like ABF, their awareness and attitudes toward the specific interventions enabling those outcomes remain largely unexplored. Future research should examine whether intervention-specific marketing claims resonate with consumers or whether outcome-based messaging remains more effective.

Longitudinal research tracking how consumer attitudes evolve over time as awareness of antimicrobial resistance and sustainable production practices increases would provide valuable insights for long-term strategic planning. Cross-cultural comparative research examining how cultural values, food traditions, and trust in institutions shape consumer responses to sustainable production claims would enhance the understanding of global market opportunities and barriers. Research examining actual purchasing behavior in real market settings, rather than hypothetical choice scenarios, would provide more reliable estimates of market potential for sustainably produced animal products. Research examining effective communication strategies for bridging the gap between technical production information and consumer understanding could inform both industry marketing and public education initiatives.

The findings from this review, combined with the identified research gaps, suggest that consumer acceptance of sustainable livestock production practices is achievable but requires continued attention to consumer education, trust building, and communication strategy development. The consistent positive attitudes toward reduced-antibiotic production documented across studies provide a foundation for market development, while the knowledge gaps and trust deficits identified represent challenges that industry and policy stakeholders must address to realize the full potential of sustainable production systems.

## 5. Conclusions

This systematic review examined peer-reviewed literature on consumer perspectives toward ABF animal products and sought to identify research specifically addressing NPIs in sustainable animal production. From 847 records, 15 studies met the inclusion criteria with seven providing empirical consumer data. The most significant finding is the identification of a critical research gap: peer-reviewed consumer research specifically examining attitudes toward named NPIs probiotics, prebiotics, phytogenics, organic acids, and enzymes as feed additives is virtually absent from the scientific literature. This finding reveals a fundamental disconnect between the substantial industry investment in developing and marketing NPIs to producers and the complete absence of knowledge about how consumers perceive or value these specific interventions. The available evidence, focused on ABF products generally, demonstrates that consumers have consistently limited knowledge of antibiotic use in animal production but hold positive attitudes toward ABF claims. One study reported WTP premiums of 18–20% for ABF chicken in Iran, though WTP quantification was limited across the evidence base. Health consciousness emerged as the strongest predictor of acceptance, while trust in certification systems and information provision significantly influenced consumer responses. For industry stakeholders, current evidence suggests that outcome-based communication like ABF resonates with consumers, while the effectiveness of intervention-specific messaging remains unknown. For policymakers, consumer acceptance of reduced-antibiotic production supports continued regulatory efforts, though clear labeling standards could enhance informed consumer choice.

## Figures and Tables

**Table 2:** Data extraction items.

Category	Data Items	Category	Data Items
Study identification	Authors; publication year; title; journal	Study context	Country/region; study setting; data collection period
Sample characteristics	Sample size; sampling method; participant demographics (age, gender, education, income); representativeness	Methodology	Study design; data collection method; measurement instruments; analytical approach
Intervention focus	Specific interventions examined; product types; comparison conditions	Outcomes	Primary and secondary outcomes; measurement methods; key findings
WTP estimates	Elicitation method; premium estimates; confidence intervals	Quality indicators	Strengths; limitations; potential biases.

**Table 3:** Quality assessment criteria.

Item	Criterion	Assessment Question	Item	Criterion	Assessment Question
Q1	Research objectives	Are research objectives clearly stated?	Q5	Measurement instruments	Are measurement instruments valid and reliable?
Q2	Study design	Is the study design appropriate for addressing objectives?	Q6	Statistical analysis	Is the statistical analysis appropriate?
Q3	Sampling strategy	Is the sampling strategy appropriate and clearly described?	Q7	Confounding	Are potential confounding factors considered?
Q4	Sample representativeness	Is the sample representative or are limitations acknowledged?	Q8	Limitations	Are study limitations acknowledged and discussed?

**Table 4:** Geographic distribution of included studies.

Region	Countries	*n*	%	Studies
Europe	Germany, UK, Netherlands, Belgium, Denmark, Sweden, Italy, Ireland	12	80.0%	[31,32,33,34,35,36,37,38,39,40,41,42]
Asia	China, Iran	3	20.0%	[24,43,44]
North America	USA	1	6.7%	[35]
Oceania	Australia	1	6.7%	[45]
Multi-country	UK/Ireland	1	6.7%	[46]
**Total**		**15**	**100.0%**	

**Table 5:** Included studies grouped by evidence type (empirical consumer studies vs. contextual reviews/modeling).

Study	Country	N	Design/Type	Product	Key Finding/Relevance
**Panel A: Empirical Consumer Studies (*n* = 7)**					
[43]	China	1123	Survey (SEM)	Meat	TPB extended model; attitude, PBC, and subjective norms affect purchase intention.
[44]	Iran	525	CVM survey	Chicken	18–20% WTP premium for ABF chicken; education and income significant.
[24]	Iran	360	Survey	Poultry	Health consciousness and food safety are key determinants.
[33]	UK	5693	Survey	Multiple meats	∼50% responded “don’t know”; limited consumer knowledge.
[46]	UK/Ireland	36	Focus groups	Meat	High AMR awareness but low understanding of transmission.
[32]	Germany	2670	Survey	Livestock products	Health risk perception; skepticism about industry responsibility.
[31]	Germany	155	Sensory + DCE	Pork (ham)	Information affects hedonic liking and WTP; organic premium.
**Panel B: Contextual Review/Modeling Articles (*n* = 8)**					
[34]	UK	–	Narrative review	–	Cattle health investment and sustainability context.
[35]	USA	–	Policy review	–	Environmental impacts of animal production (context).
[45]	Australia	–	Narrative review	–	Cattle, climate, and sustainability context.
[42]	Ireland	–	Review	–	Ruminant livestock sustainability context.
[41]	EU	–	Secondary-data analysis	Beef	Sustainable beef farming regional analysis (contextual evidence).
[36]	Netherlands	–	Modeling study	–	Livestock–feed recoupling and environmental impacts.
[38]	Norway	–	Review/analysis	–	Animal nutrition and human health gaps (contextual evidence).
[39]	Sweden	–	Economic modeling	–	Welfare-enhancing flooring economics (context/policy relevance).

Abbreviations: N = Sample Size; SEM = Structural Equation Modeling; CVM = Contingent Valuation Method; DCE = Discrete Choice Experiment; TPB = Theory of Planned Behavior; PBC = Perceived Behavioral Control; ABF = Antibiotic Free; AMR = Antimicrobial Resistance; WTP = Willingness to Pay.

**Table 6:** Quality assessment results for included empirical consumer studies (panel A).

Study	Q1	Q2	Q3	Q4	Q5	Q6	Q7	Q8
[43]	Y	Y	Y	Y	Y	Y	Y	Y
[44]	Y	Y	Y	N	Y	Y	N	Y
[24]	Y	Y	Y	N	N	Y	N	Y
[33]	Y	Y	Y	Y	Y	Y	Y	Y
[46]	Y	Y	Y	Y	N/A	N/A	N/A	Y
[32]	Y	Y	Y	Y	Y	Y	Y	N
[31]	Y	Y	N	N	Y	Y	Y	Y
**Total Met**	**7/7**	**7/7**	**6/7**	**4/7**	**5/6**	**6/6**	**4/6**	**6/7**

Abbreviations: Y = Yes (criterion met); N = No (criterion not met); N/A = Not applicable. *Color coding*: Green cells indicate that the quality criterion was met (Y), red cells indicate that the criterion was not met (N), and yellow cells indicate that the criterion was not applicable (N/A). *Quality Criteria*: Q1 = Research objectives clearly stated; Q2 = Appropriate study design; Q3 = Sampling strategy adequately described; Q4 = Sample representativeness acknowledged; Q5 = Valid and reliable measurement instruments; Q6 = Appropriate statistical analysis; Q7 = Confounding factors considered; Q8 = Study limitations discussed.

**Table 7:** WTP estimates from included studies.

Study	Product	Method	WTP Estimate	Key Moderators
[44]	Chicken	CVM	18–20% premium	Education; income; awareness of AMR
[31]	Pork (ham)	Sensory	Significant premium	Animal welfare information; organic status
[32]	Livestock products	Survey	Not quantified	Country differences; perceived health risk
[33]	Multiple meats	Survey	Not quantified	Limited knowledge; uncertainty about trade-offs
[43]	Meat	Survey	Purchase intention	Attitude; PBC; subjective norms

Abbreviations: WTP = Willingness to Pay; CVM = Contingent Valuation Method; AMR = Antimicrobial Resistance; PBC = Perceived Behavioral Control.

**Table 8:** Comprehensive synthesis of factors influencing consumer acceptance.

Category	Factor	Direction	Supporting Evidence
Health-related	Health consciousness	Strong+	Consistent predictor across studies [24,43].
	Food safety concern	+	Increases preference for ABF products [44,46].
	Risk perception	+	Higher perceived risk of antibiotics increases ABF preference [32].
	Health motivation	+	Health-motivated consumers report higher WTP [44].
Knowledge	Objective knowledge	+	Higher knowledge associated with greater acceptance [24,43].
	Subjective knowledge	Weak+	Awareness without deep understanding is common [33,46].
	Information provision	Strong+	Information significantly improves acceptance [31].
Trust	Trust in labels	+	Certification increases acceptance and WTP [31,33].
	Trust in industry	Low	Industry often perceived as insufficiently responsible [32].
	Trust in government	+	Regulatory oversight positively valued [46].
Demographics	Education	+	Consistent positive predictor [24,44].
	Income	+	Higher income enables premium purchases [44].
	Gender (female)	+	Often associated with greater health concern [33].
	Age	Mixed	Effects vary by context [40].

Abbreviations: ABF = Antibiotic Free; WTP = Willingness to Pay. Notes: Direction indicates association with consumer acceptance: + = positive association; Strong+ = consistently strong positive association across multiple studies; Weak+ = inconsistent or weak positive association; Low = generally low levels observed; Mixed = variable effects across studies.

## Data Availability

No new data were created or analyzed in this study. Data sharing is not applicable to this article.

## Supplementary Materials

The following supporting information can be downloaded at: https://www.mdpi.com/article/10.3390/ani16010070/s1. PRISMA 2020 Checklist.
