# Supplementary material for: Consumer Perspectives on Antibiotic-Free Animal Products: A Systematic Review Identifying Critical Gaps in Non-Pharmaceutical Intervention Research
Source: Animals (Basel). 2025 Dec 26;16(1):70. doi: 10.3390/ani16010070 (PMC12784898; doi:10.3390/ani16010070)
Supplement: Supplementary file 1 [file animals-16-00070-s001.zip › animals-4062226-supplementary.pdf]

PRISMA 2020 Checklist

Section and Topic	Item #	Checklist item	Location where item is reported
**TITLE**			
Title	1	Consumer Perspectives on Antibiotic-Free Animal Products: A Systematic Review Identifying Critical Gaps in Non-Pharmaceutical Intervention Research	Title page
**ABSTRACT**			
Abstract	2	Abstract follows PRISMA 2020 structure with background, objectives, methods, results, and conclusion	Page 1: Abstract follows structured PRISMA format, including background, objectives, methods, results, and conclusion.
**INTRODUCTION**			
Rationale	3	No systematic review had thoroughly compiled the literature on consumer attitudes toward those particular interventions, despite the growing significance of non-pharmaceutical interventions in sustainable animal production.	Pages 5: explains need for review linking AI, policy, prevention.
Objectives	4	This systematic review addresses this gap by pursuing the following objectives:<br>• Identify and synthesize peer-reviewed literature examining consumer perspectives toward non-pharmaceutical interventions in animal production.<br>• Characterize the geographic, methodological, and thematic scope of existing research.<br>• Assess consumer knowledge, awareness, attitudes, and WTP for products from animals raised using non-pharmaceutical interventions.<br>• Identify factors influencing consumer acceptance across demographic, psychographic, and contextual dimensions.<br>• Highlight critical gaps in the literature and establish priorities for future research.	Page 5: Section 1.1 lists the objectives clearly.
**METHODS**			
Eligibility criteria	5	The review protocol was developed a priori and specified the research questions, search strategy, eligibility criteria, data extraction procedures, quality assessment methods, and synthesis approach/	Pages 6
Information sources	6	Following PRISMA 2020 guidelines, four databases (PubMed, Web of Science, Scopus, and Google Scholar) were searched for peer-reviewed studies published from January 2020 to December 2024.	Page 1
Search strategy	7	A search string was created using terms such as consumer OR buyer OR purchaser OR shopper) AND ("antibiotic-free" OR "antimicrobial-free" OR "raised without antibiotics"	Page 6: Section 2.4
Selection process	8	Study selection followed a systematic two-stage process	Page 7: Section 2.5
Data collection process	9	Data extraction was conducted using a standardized form capturing study identifiers, methodology, sample characteristics, outcomes, and key findings.	Pages 7-8
Data items	10a	Extracted variables included study identifiers, context, sample characteristics, methodological approach, intervention focus, measurement of attitudes and WTP, and quality indicators.	Page 8: Table 2
	10b	In addition to factors unique to consumer research methods, criteria modified from the Mixed Methods Appraisal Tool (MMAT) version 2018 were used to evaluate the quality of the study.	Page 8
Study risk of bias assessment	11	Study quality was evaluated using a modified version of the Mixed Methods Appraisal Tool (MMAT 2018).	Page 8
Effect measures	12	Quantitative meta-analysis found inappropriate due to significant heterogeneity across study designs,	Page 8

Section and Topic	Item #	Checklist item	Location where item is reported
		demographics, interventions, and outcome measures.	
Synthesis methods	13a	A narrative synthesis approach was used following established guidelines	Page 9
	13b	Patterns were explored across geographical regions, demographic variables, methodological differences, and product categories.	Page 10
	13c	Results were tabulated in summary tables and displayed through thematic groupings	Page 10; Table 4
	13d	Reporting bias was not formally assessed due to variability and non-quantitative synthesis.	Page 9
	13e	No quantitative models were applied.	Page 9
	13f	Formal sensitivity analysis was not conducted.	NA
Reporting bias assessment	14	Overall, the included studies demonstrated moderate methodological quality with common limitations relating to sampling representativeness and reliance on self-reported measures.	Page 8-9
Certainty assessment	15		
**RESULTS**			
Study selection	16a	The database search identified 847 records; after removing duplicates 644 records were screened; 89 full texts were assessed and 15 studies were included in the final synthesis.	Page 7: PRISMA flow diagram
	16b	Nil	N/A
Study characteristics	17	Details of included studies, including country, sample size, methodology, and outcome measures, are summarized in Tables 4 & 5.	Pages 10–11
Risk of bias in studies	18	Overall, the included studies demonstrated moderate methodological quality with common limitations relating to sampling representativeness and reliance on self-reported measures.	Page 8-9
Results of individual studies	19	Across the included literature, consumer knowledge about antibiotic use in animal production was consistently limited, regardless of geographic setting.	Page 12
Results of syntheses	20a	Most studies were cross-sectional surveys conducted in high-income countries, with sample sizes ranging widely.	Page 10-12
	20b	Key themes included consumer knowledge, attitudes, willingness to pay (WTP), and perceived importance of animal welfare and food safety	Pages 12-18
	20c	Variability in WTP outcomes was linked to country, income, and familiarity with antibiotic use in livestock.	Pages 13-16
	20d	No formal statistical sensitivity analysis was conducted.	Page 9
Reporting biases	21	Reporting bias was not assessed due to the heterogeneity and qualitative synthesis.	Page 9
Certainty of evidence	22	No formal certainty assessment conducted.	N/A
**DISCUSSION**			
Discussion	23a	The review identifies a critical gap: the absence of consumer-focused research specifically addressing non-pharmaceutical interventions such as probiotics, prebiotics, phytogenics, organic acids, and enzymes.	Pages 16-17
	23b	The evidence base remains geographically concentrated and methodologically heterogeneous, limiting broad generalizability.	Page 18
	23c	The review relied primarily on four major databases and English-language publications.	Page 18

PRISMA 2020 Checklist

Section and Topic	Item #	Checklist item	Location where item is reported
	23d	Findings underscore the need for targeted communication strategies, policy development, and expanded consumer research in low- and middle-income settings.	Pages 18-20
**OTHER INFORMATION**			
Registration and protocol	24a	Not registered	N/A
	24b	Nil	N/A
	24c	Nil	N/A
Support	25	This research was supported by the Ministry of Education and Ministry of Science & ICT, Republic of Korea (grant numbers: NRF [2021-R1-I1A2 (059735)], RS [2024-0040 (5650)], RS [2024-0044 (0881)], RS [2019-II19 (0421)], and RS [2025-2544 (3209)].	Funding section
Competing interests	26	No conflicts declared	No conflicts declared
Availability of data, code and other materials	27	All data extracted	Not reported

From: Page MJ, McKenzie JE, Bossuyt PM, Boutron I, Hoffmann TC, Mulrow CD, et al. The PRISMA 2020 statementreporting systematic reviews. BMJ 2021;372:n71. doi: 10.1136/bmj.n71. This work is licensed under CC BY 4.0. To view a copy of this license, visit https://creativecommons.org/licenses/by/4.0/
